# Acute Influenza Virus-Associated Encephalitis and Other Neurological Complications in Severe Hospitalized Laboratory-Confirmed Influenza Cases—Catalonia 2010–2020

**DOI:** 10.3390/pathogens14030237

**Published:** 2025-02-28

**Authors:** Pilar Ciruela, Nuria Soldevila, Nuria Torner, Luca Basile, Maria del Mar Mosquera, M. Angeles Marcos, Anna Martínez, Mireia Jané, Cristina Rius, Angela Domínguez, the Working Group for the Catalan Influenza and Severe Acute Respiratory Infection Sentinel Surveillance Network (PIDIRAC)

**Affiliations:** 1 CIBER Epidemiology and Public Health CIBERESP, Instituto de Salud Carlos III, 28029 Madrid, Spain; pilar.ciruela@gencat.cat (P.C.); a.martinez@gencat.cat (A.M.); mireia.jane@gencat.cat (M.J.); crius@aspb.cat (C.R.); angela.dominguez@ub.edu (A.D.); 2 Public Health Agency of Catalonia, 08005 Barcelona, Spain; luca.basile_ext@gencat.cat; 3 Department of Medicine, University of Barcelona, 08036 Barcelona, Spain; mdmosquera@clinic.cat; 4 Department of Microbiology, Hospital Clínic of Barcelona-ISGLOBAL, 08036 Barcelona, Spain; mmarcos@clinic.cat; 5 CIBER de Enfermedades Infecciosas CIBERINFEC, Instituto Salud Carlos III, 28029 Madrid, Spain; 6 Public Health Agency of Barcelona, 08023 Barcelona, Spain

**Keywords:** influenza, encephalitis, neurological complication

## Abstract

Neurological complications associated with influenza (NCIs) are rare events in adults. Influenza-associated encephalopathy is one of the most severe and frequently reported NCIs. The aim of this study is to describe the frequency and characteristics of NCIs in adults during 10 post-2009 pandemic influenza seasons. Data were obtained from the registry of influenza cases admitted to hospitals of the PIDIRAC network for the surveillance of severe hospitalized laboratory-confirmed influenza (SHLCI) cases in Catalonia from October 2010 to March 2020. The variables analyzed were NCI, age, antiviral treatment, vaccination status, and outcome at discharge. During the study period, 9 (1.5‰) of 5931 SHLCI cases presented NCI. Five (55.6%) had influenza A and four (44.4%) had influenza B. Median age was 62 (17–67) years. One case had been vaccinated, all had received antiviral treatment, and five required ICU admission. The mean length of stay was 25.6 days (SD 25.8). Encephalitis was the most frequent complication, occurring in six cases (66.7%). Of these, three cases (50%) were caused by influenza A (two AH1N1pdm09 strains and one AH3N2). The high frequency of influenza-associated encephalitis caused by both type A and B influenza viruses suggests that both should be considered as potential etiologic factors for encephalopathy and other neurological diseases in adults. This recommendation would allow for the prompt antiviral treatment and prevention of severe outcomes.

## 1. Introduction

Influenza is a highly infectious disease caused by a single-stranded RNA virus and is a significant cause of illness and death, particularly during seasonal outbreaks. Influenza virus primarily affects the respiratory system, being the most frequent cause of acute upper respiratory tract infections, especially in the winter season [1]. Although most patients recover completely from influenza, there is a certain proportion of patients at risk of complications that require hospitalization. The most frequent severe complications of influenza affect the respiratory tract and include pneumonia (primary or secondary to bacterial infection), bronchitis, and exacerbations of chronic pulmonary diseases. Additionally, the influenza virus has a neurotropic potential that can cause short- and long-term consequences across the central nervous system (CNS) [2]. The most commonly encountered extra-respiratory complication is encephalitis, which usually occurs 1 week following the first symptoms of influenza [3,4]. Encephalitis, characterized by inflammation of the brain parenchyma, is most commonly caused by viruses. Herpes viruses remain the most common causes of sporadic encephalitis, with herpes simplex virus type 1 and varicella zoster virus reported most frequently. In endemic regions, arboviruses such as Japanese encephalitis virus and West Nile virus contribute significantly to the disease burden. Importantly, up to two-thirds of survivors are left with substantial long-term neurological complications, particularly neurocognitive impairment [5,6].

Influenza-related encephalitis is a rapidly progressive encephalopathy that usually presents in the early phase of influenza infection and primarily induces central nervous system dysfunction. The pathogenesis of influenza virus-induced CNS disease in humans is largely unknown. It is possibly caused by an indirect effect due to systemic cytokines, or by a direct effect of virus entry into the CNS [2].

A spectrum of neurologic complications associated with influenza (NCI) virus infection has been recognized and includes encephalopathy, seizures, and Guillain–Barré syndrome (GBS). Similarly, influenza AH1N1pdm09 virus infection has been associated with neurologic manifestations, but information about the spectrum of neurologic complications and burden of disease has been limited to case reports and small case series [7].

Encephalitis, ataxia, and seizures were the most prevalent encephalopathy-related disorders. The influenza A H1N1 pandemic in 2009 proved to be more neurotoxic, especially in children under 5 years old [8]. NCIs are rare events in adults with seasonal influenza. Information about the characteristics of neurological complications and the burden of disease has been limited to case reports, mainly during the 2009 pandemic. Although most neurological complications are temporary, permanent sequelae and death can also occur. Being a relatively uncommon severe complication following influenza, neurological manifestations may be overlooked by clinicians and knowledge is limited about the frequency, diagnosis, treatment, and prognosis of NCI. Although influenza-associated encephalitis is rare, it is a serious neurological complication that has been mostly described in children. To our knowledge there is a certain lack of publications that deal with an adult population, making large-scale epidemiological data limited. This study aimed to conduct a retrospective study of the frequency and the characteristics of NCI in adult severe hospitalized laboratory-confirmed influenza (SHLCI) virus infection during 10 post-pandemic 2009 influenza seasons.

## 2. Materials and Methods

Information regarding NCI was gathered from hospital registries from influenza cases admitted to hospitals included in the PIDIRAC network for the surveillance of severe hospitalized laboratory confirmed influenza cases in Catalonia from October 2010 until March 2020.

The variables analyzed were age, influenza virus type and subtype, antiviral treatment, vaccination status, intensive care unit (ICU) admission, length of hospital stay, and outcome at discharge. Proportions, median, mean, and standard deviation were calculated to describe the characteristics of NCI. Analyses were performed using the IBM^®^ SPSS^®^ (Armonk, NY, USA) v.29 statistical package.

## 3. Results

**Figure 1:**
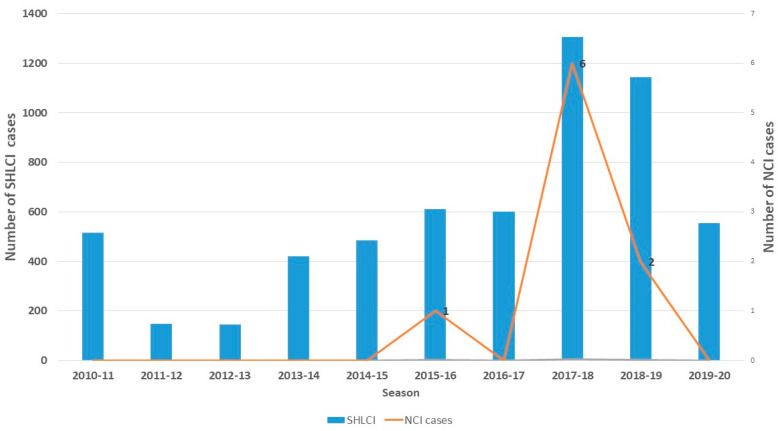
Severe hospitalized laboratory-confirmed influenza (SHLCI) and number of cases with neurological complications associated to influenza (NCIs). Influenza and acute respiratory infection surveillance program (PIDIRAC), Catalonia, 2010–2020.

The PIDIRAC surveillance of severe confirmed influenza cases that required hospital admission registered a total of 5931 cases during 10 influenza epidemic seasons (2010–2020). Of these, five seasons had AH1N1-predominant influenza virus circulation, two AH3N2, two B, and one mixed AH1N1 and AH3N2. Seasons 2017–2018 and 2018–2019 were outstanding because of the great number of SHLCI cases registered (1306 and 1145, respectively), as shown in Figure 1.

Consequently, these two seasons presented the higher proportion of NCIs (4.6‰ and 1.7‰ of cases, respectively).

Thus, during the study period, 9 (1.5‰) of the 5931 patients admitted with laboratory-confirmed influenza presented NCIs. Five cases (55.6%) had influenza A (H3 and H1N1_pdm09_) and four cases (44.4%) had influenza B, as shown in Table 1.

**Figure 2:**
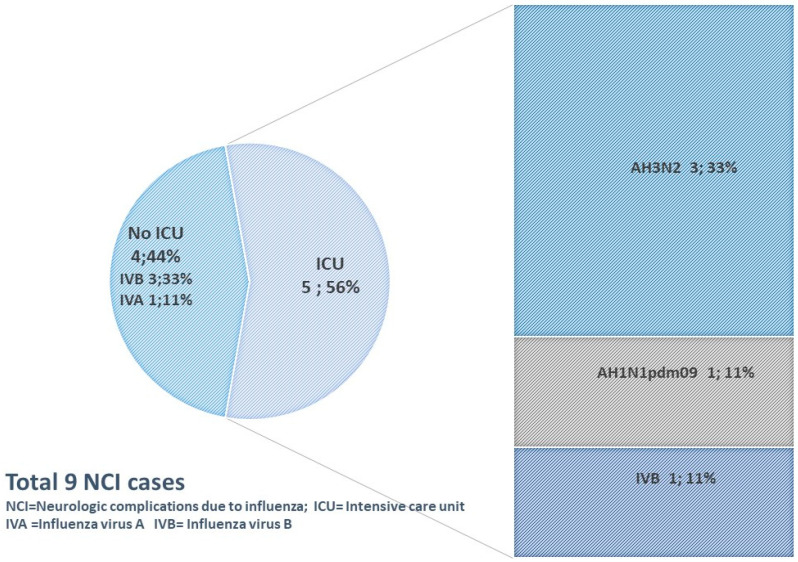
Intensive care unit (ICU) admission of severe hospitalized laboratory-confirmed influenza (SHLCI) cases with neurological complications, according to influenza virus type and subtype. Influenza and acute respiratory infection surveillance program (PIDIRAC), Catalonia, 2010–2020.

The median age of the cases was 62 (range 17–67 years). Regarding vaccination status, only one case had received that season’s influenza vaccine. All had received antiviral treatment upon admission. Five cases required ICU admission (four had influenza A and one influenza B; Figure 2) and the mean length of stay was 25.6 days (SD 25.8). Encephalitis was the most frequent complication diagnosed in six (66.7%) patients, of whom three (50%) had influenza A (two had H1N1pdm09 and one had H3N2). Moreover, three (33%) had GBS, meningitis, and myelitis, respectively. No death nor permanent sequels were observed.

## 4. Discussion

There is a wide span of viruses that cause neurological impairment, such as West Nile virus, Japanese encephalitis virus, Toscana virus, and many more including influenza viruses [2]. Influenza A viruses (H3N2 and H1N1pdm09) circulate within the population, causing yearly epidemics and the most common extra-respiratory complication of influenza is the development of central nervous system disease [9]. Influenza virus infections have been linked to a wide array of neurological diseases such as febrile seizures, meningitis and encephalitis, or the development of GBS.

In our study, albeit with few cases recorded during the 10 epidemic seasons studied, encephalitis was the most frequent neurological complication.

Among the SHLCI cases, an incidence of 1.5‰ for NCIs was observed. As reported by several authors, some strains are more frequently associated with CNS disease [8,10,11] and in our study influenza B infection showed a higher frequency of neurological complication outcomes. This might be caused by the greater incidence of severe infections among the elderly population during the 2017–2018 season with a predominance of influenza B virus circulation [12,13]. According to a systematic review on influenza-related encephalopathy after 2000, carried out by Zhang et al., the AH1N1pdm09 influenza outbreak in 2009 played a major role in research on influenza-related encephalopathy since 2010 [7]. A major limitation of the study is the small number of cases registered during the ten-year period, although the findings improve knowledge of extra-pulmonary complications in severe influenza. Furthermore, in our study, in spite of the small number of cases, there was a higher predominance of influenza B virus infections related to neurological conditions. The higher frequency of influenza-associated encephalopathy/encephalitis caused by type B influenza viruses warrants considering both influenza A and B as etiologic factors of encephalopathy and other neurological diseases in adults hospitalized for severe influenza infection. This recommendation would allow for prompt antiviral treatment and the prevention of severe outcomes. With the irruption of the COVID-19 pandemic in late 2019 and early 2020, and with it being an infectious respiratory disease that shares routes and means of transmission, clinical characteristics and outcomes, and laboratory and radiological manifestations with the influenza virus, the overlap of SARS-CoV-2 and the influenza virus during winter can lead to co-infections [14]. SARS-CoV2 infection can also cause neurological disease as a result of virus invasion with an early onset of neurological symptoms [15]. This fact highlights the importance of prevention by immunization and the early treatment of acute respiratory infections such the COVID-19 and influenza viruses. Influenza vaccination can reduce disease severity and this additional benefit of influenza vaccination on disease severity includes frequent and less frequent complications, such as those affecting CNS [16]. The post-pandemic co-circulation of both viruses and the lack of non-pharmaceutical interventions implemented during the COVID-19 pandemic that restrained the circulation of influenza viruses [17], renders research on influenza-related and SARS-CoV-2 encephalopathy crucial, especially considering that its high-risk population is children and the elderly with a risk of brain injury sequelae. This study highlights the performance of the influenza B virus as a cause of encephalitis and the importance of prevention for influenza-related severe outcomes, including neurological complications, through seasonal influenza vaccination.

## Figures and Tables

**Table 1:** Distribution of severe hospitalized laboratory-confirmed influenza and neurological complications associated to influenza by type/subtype and influenza season. Influenza and acute respiratory infection surveillance program (PIDIRAC), Catalonia, 2010–2020.

Influenza Season	2010–2011	2011–2012	2012–2013	2013–2014	2014–2015	2015–2016	2016–2017	2017–2018	2018–2019	2019–2020
Predominant Influenza Virus Type/Subtype	AH1N1 pdm09	AH3N2	B	AH1N1 pdm09	AH3N2/AH1N1 pdm09	AH1N1 pdm09	AH3N2	B	AH1N1 pdm09	AH1N1 pdm09
Number of SHLCI * cases	516	148	145	420	484	612	601	1306	1145	554
NCI ** cases	-	-	-	-	-	1	-	6	2	-
**Cases/1000**	-	-	-	-	-	1.63	-	4.59	1.75	-

* SHLCI: severe hospitalized laboratory-confirmed influenza; ** NCI: neurological complications associated to Influenza.

## Data Availability

The data are available upon request to the corresponding authors.

## Abbreviations

The following abbreviations are used in this manuscript:

NCI	Neurological complications associated to influenza
IAE	Influenza-associated encephalopathy
ICU	Intensive care unit
IVA	Influenza virus A
IVB	Influenza virus B
SHLCI	Severe hospitalized laboratory-confirmed influenza
GBS	Guillain–Barré syndrome

## References

[1] Boktor S.W., Hafner J.W. (2025). Influenza.

[2] Ludlow M., Kortekaas J., Herden C., Hoffmann B., Tappe D., Trebst C., Griffin D.E., Brindle H.E., Solomon T., Brown A.S. (2016). Neurotropic Virus Infections as the Cause of Immediate and Delayed Neuropathology. Acta Neuropathol..

[3] Sellers S.A., Hagan R.S., Hayden F.G., Fischer W.A. (2017). The Hidden Burden of Influenza: A Review of the Extra-Pulmonary Complications of Influenza Infection. Influenza Other Respir. Viruses.

[4] Paksu M.S., Aslan K., Kendirli T., Akyildiz B.N., Yener N., Yildizdas R.D., Davutoglu M., Yaman A., Isikay S., Sensoy G. (2018). Neuroinfluenza: Evaluation of Seasonal Influenza Associated Severe Neurological Complications in Children (a Multicenter Study). Child’s Nerv. Syst..

[5] Duerlund L.S., Nielsen H., Bodilsen J. (2024). Current Epidemiology of Infectious Encephalitis: A Narrative Review. Clin. Microbiol. Infect..

[6] Kvam K.A., Stahl J.-P., Chow F.C., Soldatos A., Tattevin P., Sejvar J., Mailles A. (2024). Outcome and Sequelae of Infectious Encephalitis. J. Clin. Neurol..

[7] Zhang Z., Tan J., Li Y., Zhou X., Niu J., Chen J., Sheng H., Wu X., Yuan Y. (2023). Bibliometric Analysis of Publication Trends and Topics of Influenza-related Encephalopathy from 2000 to 2022. Immun. Inflamm. Dis..

[8] Glaser C.A., Winter K., DuBray K., Harriman K., Uyeki T.M., Sejvar J., Gilliam S., Louie J.K. (2012). A Population-Based Study of Neurologic Manifestations of Severe Influenza A(H1N1)Pdm09 in California. Clin. Infect. Dis..

[9] Kuiken T., Taubenberger J.K. (2008). Pathology of Human Influenza Revisited. Vaccine.

[10] Bailey H.E. (2020). Acute Necrotizing Encephalopathy Associated with Influenza A. Neurodiagn. J..

[11] Dou Y., Li Y. (2022). Influenza A H3N2-Associated Meningoencephalitis in an Older Adult with Viral RNA in Cerebrospinal Fluid: Case Report. Front. Neurol..

[12] Soldevila N., Basile L., Martínez A., Torner N., Marcos M.Á., Mosquera M.d.M., Antón A., Andrés C., Rius C., Pumarola T. (2022). Surveillance of Influenza B Severe Hospitalized Cases during 10 Seasons in Catalonia: Does the Lineage Make a Difference?. J. Med. Virol..

[13] Basile L., Torner N., Martínez A., Mosquera M.M., Marcos M.A., Jane M. (2019). Seasonal Influenza Surveillance: Observational Study on the 2017–2018 Season with Predominant B Influenza Virus Circulation. Vacunas.

[14] Osman M., Klopfenstein T., Belfeki N., Gendrin V., Zayet S. (2021). A Comparative Systematic Review of COVID-19 and Influenza. Viruses.

[15] Stoian A., Bajko Z., Stoian M., Cioflinc R.A., Niculescu R., Arbănași E.M., Russu E., Botoncea M., Bălașa R. (2023). The Occurrence of Acute Disseminated Encephalomyelitis in SARS-CoV-2 Infection/Vaccination: Our Experience and a Systematic Review of the Literature. Vaccines.

[16] Arriola C., Garg S., Anderson E.J., Ryan P.A., George A., Zansky S.M., Bennett N., Reingold A., Bargsten M., Miller L. (2017). Influenza Vaccination Modifies Disease Severity Among Community-Dwelling Adults Hospitalized With Influenza. Clin. Infect. Dis..

[17] Ye Q., Liu H., Mao J., Shu Q. (2023). Nonpharmaceutical Interventions for COVID-19 Disrupt the Dynamic Balance between Influenza A Virus and Human Immunity. J. Med. Virol..

